# Diabetes propels the risk for cardiovascular disease: sweet monocytes becoming aggressive?

**DOI:** 10.1007/s00018-016-2316-9

**Published:** 2016-07-28

**Authors:** Janna A. van Diepen, Kathrin Thiem, Rinke Stienstra, Niels P. Riksen, Cees J. Tack, Mihai G. Netea

**Affiliations:** 1 Department of Internal Medicine, Radboud Institute for Molecular Life Sciences, Radboud University Nijmegen Medical Center, Radboudumc, (463), P. O. Box 9101, 6500 HB Nijmegen, The Netherlands; 2 Nutrition, Metabolism and Genomics Group, Division of Human Nutrition, Wageningen University, 6703 HA Wageningen, The Netherlands; 3 Radboud Center for Infectious Diseases, Radboud University Nijmegen Medical Center, 6525 GA Nijmegen, The Netherlands

**Keywords:** Diabetes, Hyperglycemia, Atherosclerosis, Trained immunity, Inflammation, Epigenetics, Metabolism

## Abstract

Diabetes strongly predisposes to cardiovascular disease (CVD), the leading cause of mortality in these patients, as well as in the entire population. Hyperglycemia is an important cardiovascular risk factor as shown by the observation that even transient periods of hyperglycemia, despite return to normoglycemia during follow-up, increase the risk for CVD, a phenomenon termed ‘hyperglycemic memory’. The molecular mechanisms underlying this phenomenon remain largely unknown. As inflammation plays an important role in the pathogenesis of atherosclerosis, we propose that long-term functional reprogramming of monocytes and macrophages, induced by hyperglycemia, plays an important role in the phenomenon of hyperglycemic memory leading to cardiovascular complications in patients with diabetes. In this review, we discuss recent insights showing that innate immune cells possess the capacity to reprogram their function through epigenetically mediated rewiring of gene transcription, a process termed ‘trained immunity’. The long-term reprogramming of monocytes can be induced by microbial as well as metabolic products, and involves a shift in cellular metabolism from oxidative phosphorylation to aerobic glycolysis. We hypothesize that hyperglycemia in diabetes patients induces long-term activation of monocytes and macrophages through similar mechanisms, thereby contributing to plaque development and subsequent macrovascular complications.

## Introduction

There are 422 million people with diabetes worldwide and probably many more individuals with undiagnosed diabetes. The presence of diabetes severely augments the risk for atherosclerosis and associated cardiovascular diseases (CVD), making cardiovascular disease the leading cause of morbidity and mortality in individuals diagnosed with type 1 diabetes (T1D) or type 2 diabetes mellitus (T2D), as well as the entire population. Multiple risk factors have been described to contribute to diabetic complications, such as genetic predisposition [1], dyslipidemia [2], and hypertension [3], which cause proatherogenic processes in diabetes patient’s independent of hyperglycemia. Nonetheless, several prospective studies have revealed that hyperglycemia per se, a defining characteristic of diabetes, is an important and independent risk factor for cardiovascular disease in patients with T1D and T2D [4–6]. Indeed, among individuals with diabetes, a 1 % rise in hemoglobin A1c (HbA1c) levels is associated with a 31 % increase in cardiovascular events [7].

The importance of hyperglycemia as a risk factor is also demonstrated by the observation that a period of elevated HbA1c levels in the past translates into a higher future incidence of CVD despite return to normoglycemia during follow-up [4, 8]. Thus, the Diabetes Control and Complications Trial and Epidemiology of Diabetes Interventions and Complications (DCCT/EDIC) study have played a pivotal role in shaping our understanding of glycemic control. Indeed, the DCCT showed that intensive glucose-lowering therapy strongly reduced HbA1c levels compared with the conventional therapy. After a mean follow-up of 6.5 years, the cohort was subsequently transferred to the EDIC trial, where all patients were encouraged to intensive glucose-lowering therapy and within 1 year, glycemic control had become similar in both groups. Unexpectedly, and thus, despite almost equal glycemic control during the EDIC follow-up, the original conventional therapy group continued to develop microvascular and macrovascular complications at a higher rate than the group initially assigned intensive therapy. This persistent risk for complications observed for T1D patients in the DCCT/EDIC trial has also been found in T2D patients in the United Kingdom Prospective Diabetes Study (UKPDS) trial and subsequently been defined as ‘hyperglycemic memory’ or the ‘legacy effect’ [8, 9]. A growing body of evidence now suggests that adverse effects of prior hyperglycemia are ‘memorized’ by vascular cells or tissues over time, translating into an enhanced risk for CVD. The molecular mechanisms responsible for this hyperglycemic memory are still far from understood, although epigenetic mechanisms are thought to underlie this phenomenon [10, 11].

Epigenetic modifications refer to modifications in chromatin state or non-coding RNAs that may lead to long-term reprogramming in gene expression without changing the nucleotide sequence of the DNA itself. Prior episodes of hyperglycemia associate with epigenetic changes in various vascular cell types in vitro and ex vivo (i.e., smooth muscle cells and endothelial cells) conferring cell memories and may be of critical importance in the development and progression of vascular complications, as reviewed elsewhere [11–13]. It has only been recently shown that monocytes and macrophages, guardians of non-specific host defense, can also build up long-term memory via epigenetic reprogramming, a process termed ‘trained immunity’ [14, 15]. Innate immune cells are major regulators of atherosclerotic plaque development and progression [16]. Therefore, insights into mechanisms controlling (long-term) activation of innate immune cells, especially in diabetes patients, are of crucial importance to understanding persistent vascular complications.

In the current review, we address the current knowledge on the activation of monocytes and macrophages in patients with diabetes in general and by hyperglycemic conditions specifically. Subsequently, we discuss recent novel insights into mechanisms implicated in long-term activation and memory of monocytes and macrophages, defined as trained immunity. We finally propose that reprogramming of monocytes by hyperglycemia may contribute to the hyperglycemic memory as an inducer of vascular complications in patients with diabetes.

## Hyperglycemia associates with monocyte activation to contribute to the pathogenesis of atherosclerosis

Initially viewed as primarily the result of lipid accumulation, atherosclerotic development is currently considered as a low-grade inflammatory disease of the arterial wall. Altered monocyte and macrophage numbers, function, and skewed proinflammatory signaling are considered to facilitate vascular inflammation [16]. In the process of atherosclerotic plaque formation, circulating monocytes bind to activated endothelial cells, and transmigrate into the subendothelial space of the arterial wall where maturation into macrophages occurs [17]. Macrophages express scavenger receptors for uptake of modified lipoproteins leading to foam cell formation. In addition, resident macrophages are major contributors to the local inflammatory response through secretion of proinflammatory mediators, including chemokines, cytokines, and reactive oxygen species (ROS) [16]. Both macrophage numbers and their inflammatory phenotype influence plaque fate [16].

### Circulating monocyte numbers and function in diabetes patients

In high-risk populations, the amount of circulating monocytes independently predicts the risk for coronary artery disease [18–20]. In diabetes subjects, monocyte counts are increased in T2D, but not T1D patients [21, 22]. It would be interesting to investigate whether subsets of monocytes are affected in T1D patients, especially because animal studies show that hyperglycemia specifically increases inflammatory Ly6-C^hi^ monocytes, whereas non-inflammatory Ly6-C^lo^ monocytes were largely unaffected [23].

Functional alterations have been observed in monocytes isolated from both T2D and T1D patients. Monocyte activation in T2D patients has been associated ex vivo with enhanced inflammasome activation, known to activate caspase-1 ultimately leading to proteolytic cleavage and activation IL-1β and IL-18 [24]. Indeed, monocytes of T2D patients also show increased proinflammatory interleukin (IL)-1β and ROS secretion upon activation [25, 26], as well as enhanced adhesion to the endothelium [26]. T2D is frequently accompanied by multiple cardiovascular risk factors, such as obesity, dyslipidemia and/or high blood pressure, which also affect monocyte number and function [27]. Therefore, to investigate the direct relation between hyperglycemia and monocyte activation, findings in T1D patient are easier to interpret. Monocytes from T1D patients show increased release of proinflammatory cytokines IL-6, IL-1β, and tumor necrosis factor α (TNFα) after ex vivo activation of monocytes by interferon γ (IFNγ) or lipopolysaccharide (LPS) [28, 29]. In addition, binding of monocytes to cultured endothelial cells has been shown to be increased in T1D patients [30], although this could not be confirmed by others [28]. Importantly, the adhesion of monocytes to endothelial cells may depend on the level of hyperglycemia, since monocytes derived from poorly controlled diabetes patients show higher binding to endothelial cells than monocytes from well-controlled patients [31] implying an important role of glucose in activation of innate immune cells.

### Relevance for CVD

The proinflammatory phenotype of monocytes with increased binding to endothelium is expected to increase monocyte migration in atherosclerotic plaques [32]. Autopsy studies indeed reveal that plaques from patients with T1D and T2D are characterized by enhanced macrophage infiltration [33]. Interestingly, plaque macrophage content was associated with HbA1c levels, independently of any other risk factors (e.g., plasma cholesterol levels), suggesting a direct link between hyperglycemia and plaque macrophage infiltration [33]. A recent study investigated expression of proinflammatory and anti-inflammatory cytokines in both monocytes as well as atherosclerotic plaque material derived from T2D patients undergoing carotid endarterectomy. Despite a somewhat underpowered study design, the authors reported a positive correlation between HbA1c levels and proinflammatory signaling in both circulating monocytes as well as atherosclerotic plaques, as determined by an increased ratio between proinflammatory IL-6 and TNFα versus anti-inflammatory IL-10 expression [34].

### Hyperglycemia and monocyte activation

Experimental murine and in vitro cell culture studies have shed more light on possible mechanisms that may explain the effect of hyperglycemia on monocyte activation. Peritoneal macrophages isolated from T1D and T2D mouse models show increased proinflammatory cytokine expression [35]. Recent studies in animals show that glucose itself enhances diabetes-associated atherosclerosis by promoting proliferation and proinflammatory responses of innate immune cells and their bone marrow progenitors [23]. Multiple in vitro studies have revealed that high glucose leads to increased expression of inflammatory cytokine genes in primary-derived or cell line-derived monocytes and macrophages [36–38], as well as increased binding of monocytes to endothelial cells [37]. Importantly, although the transcriptional levels of proinflammatory mediators can be measured in cells exposed to high glucose levels [37], an actual increased secretion of inflammatory cytokines only becomes apparent after costimulation, e.g., with phorbol 12-myristate 13-acetate (PMA) [36], LPS or *Mycobacterium tuberculosis* [39, 40]. This suggests that glucose itself only ‘primes’ monocytes, by inducing (mild) changes in the transcriptional program, while a clear proinflammatory phenotype only evolves after costimulation with pathogenic stimuli. Perhaps, monocytes previously exposed to high glucose levels develop a similar proinflammatory phenotype after restimulation with pathogenic stimuli, indicating a memorization of hyperglycemic events.

Thus, there is strong evidence that chronic hyperglycemia, the hallmark of diabetes, is associated with a (low-grade) activation of the innate immune system, which, in turn, is associated with accelerated atherosclerosis. The phenomenon of hyperglycemic memory, i.e., the observation that even transient periods of hyperglycemia enhance development of atherosclerosis, suggests that elevated glucose may be somehow memorized and induce long-term activation of the innate immune system, which eventually accelerates atherosclerosis development. However, knowledge on the molecular mechanisms controlling these processes is lacking. Identifying these pathways may help in our efforts to find new therapeutic targets to treat or prevent atherosclerosis.

## The concept of trained immunity in the pathogenesis of atherosclerosis

Recent discoveries in the field of innate immunity may shed light on how monocytes and macrophages adopt a long-term proinflammatory phenotype in patients with risk factors for atherosclerosis. In recent years, it has been revealed that innate immune cells exhibit an immunological (non-specific) memory of past insults, which has been named “trained immunity” or “innate immune memory” [14, 15]. With respect to host defense toward pathogens, immune responses in vertebrates were classically divided into innate (e.g., monocytes and macrophages) and adaptive (B- and T-cells), with only the latter being able to build specific immunological memory. However, this dogma has been challenged with the discovery that monocytes possess a non-specific long-term memory that changes their functional program, resulting in protection from reinfection independent of T and B lymphocytes [41, 42]. Functionally, this means that priming of innate immune cells with an initial (microbial) challenge results in an enhanced responsiveness to a secondary challenge [14]. This enhanced responsiveness of monocytes is characterized by increased production of inflammatory mediators. Mechanistic studies showed that the reprogramming of monocytes is mediated by epigenetic modifications [14, 42–44]. Importantly, these changes allow for an increased response to restimulation of cells through both the same and different pattern recognition receptors (PRRs), and can persists for weeks to months [14]. So far, monocyte training with fungal cell wall β-glucans, *Candida* or Bacillus Calmette–Guérin (BCG) is associated with changes in H3K4me3 at promoters of proinflammatory cytokines [42], as well as changes in other chromatin marks, such as H3K4me1, H2K27Ac, and H3K9me2 [43, 45]. In addition to these chromatin marks, other additional histone modifications or DNA methylation changes are likely to be involved in the process of trained immunity, but have not yet been identified.

Although this phenomenon of trained immunity can be beneficial in the context of infections, long-term activation of the innate immune system might be detrimental in chronic inflammatory diseases including atherosclerosis [46]. Importantly, microbial ‘training’ of monocytes activates transcriptional programming of various genes involved in the process of atherosclerosis, including proinflammatory cytokines, chemokines, and scavenger receptors, which results in increased foam cell formation [47, 48]. This implies that circulating monocytes that have not yet infiltrated the plaque are exposed to environmental signals that may induce epigenetic reprogramming of the cells, which, in turn, makes them more responsive toward a second stimulus, e.g., within the atherosclerotic plaques. Importantly, the induction of trained immunity is not restricted to microbial products, since oxidized LDL (oxLDL) can also induce trained immunity and long-term atherosclerotic properties of monocytes via similar epigenetic modifications in vitro [48]. The training of monocytes by oxLDL implies that monocytes previously exposed in circulation are particularly prone to develop into proinflammatory cells, or foam cells, once entering the arterial wall. The training efficacy of other endogenous compounds associated with metabolic diseases, including cholesterol crystals, free fatty acids (FFAs), and advanced glycation end products (AGEs), remains unknown. Importantly, these compounds are known to induce an (low-grade) inflammatory response in monocytes via activation of various PRRs [49–51]. Whether they additionally have the potential to epigenetically reprogram monocytes and, thereby, modify their long-term (proinflammatory) behavior, remains to be established [47].

Clinical evidence for the concept of trained immunity in coronary artery disease has been recently provided by the study of Shirai et al. This study evaluated whether monocytes derived from patients with coronary artery disease are primed differently, resulting in proinflammatory cytokine production upon entering the tissue space, by comparing ex vivo-differentiated macrophages from patients and controls [52]. Patient-derived monocytes showed increased production of proinflammatory cytokines IL-6 and IL-1β after stimulation, which persisted once cells differentiated into macrophages, suggesting a different reprogramming of the cells. Further studies are required to determine the role of chromatin modifications in this persistent gene expression.

Collectively, the long-term memory of innate immune cells mediated by epigenetic reprogramming has important implications for our understanding of the pathogenesis of atherosclerosis, although solid clinical evidence is still needed. In addition, further studies are required to evaluate the concept of trained immunity in response to other metabolites associated with metabolic disease. AGEs and high glucose levels that are increased in patients with diabetes represent interesting candidates to evaluate whether trained immunity could be an underlying mechanisms involved in the phenomenon of hyperglycemic memory.

## Can trained immunity contribute to atherosclerosis in diabetes patients?

The identification of oxLDL as an inducer of trained immunity suggests a role for metabolic endogenous compounds in training of monocytes, potentially unraveling epigenetic reprogramming of monocytes as a novel pathogenic pathway contributing to atherosclerotic development. Importantly, this opens new avenues for future research to explore the contribution of trained immunity to the phenomenon of hyperglycemic memory in patients with diabetes. Shirai et al. revealed that various risk factors for coronary events, i.e., hypertension, hyperlipidemia, and T2D all correlated with IL-6 release by monocytes ex vivo, showing considerable potential for exploration of other atherogenic risk factors that could induce training of monocytes [52]. A previous study showed that monocytes obtained from T2D patients displayed enhanced inflammasome activation [24]. Moreover, after differentiation to macrophages ex vivo, cells from T2D patients showed an enhanced cytokine secretion in response to the bacterial components LPS and Pam3Cys [24]. This study implies that the diabetic milieu reprograms the function of circulating monocytes that have not yet infiltrated the atherosclerotic plaque, which may lead to a more proinflammatory phenotype after differentiation or ex vivo culture. Hence, translating these ex vivo observations to the in vivo situation, reprogrammed monocytes recruited to the atherosclerotic plaques may develop into more proatherogenic macrophages and accelerate the development of CVD.

**Fig. 1:**
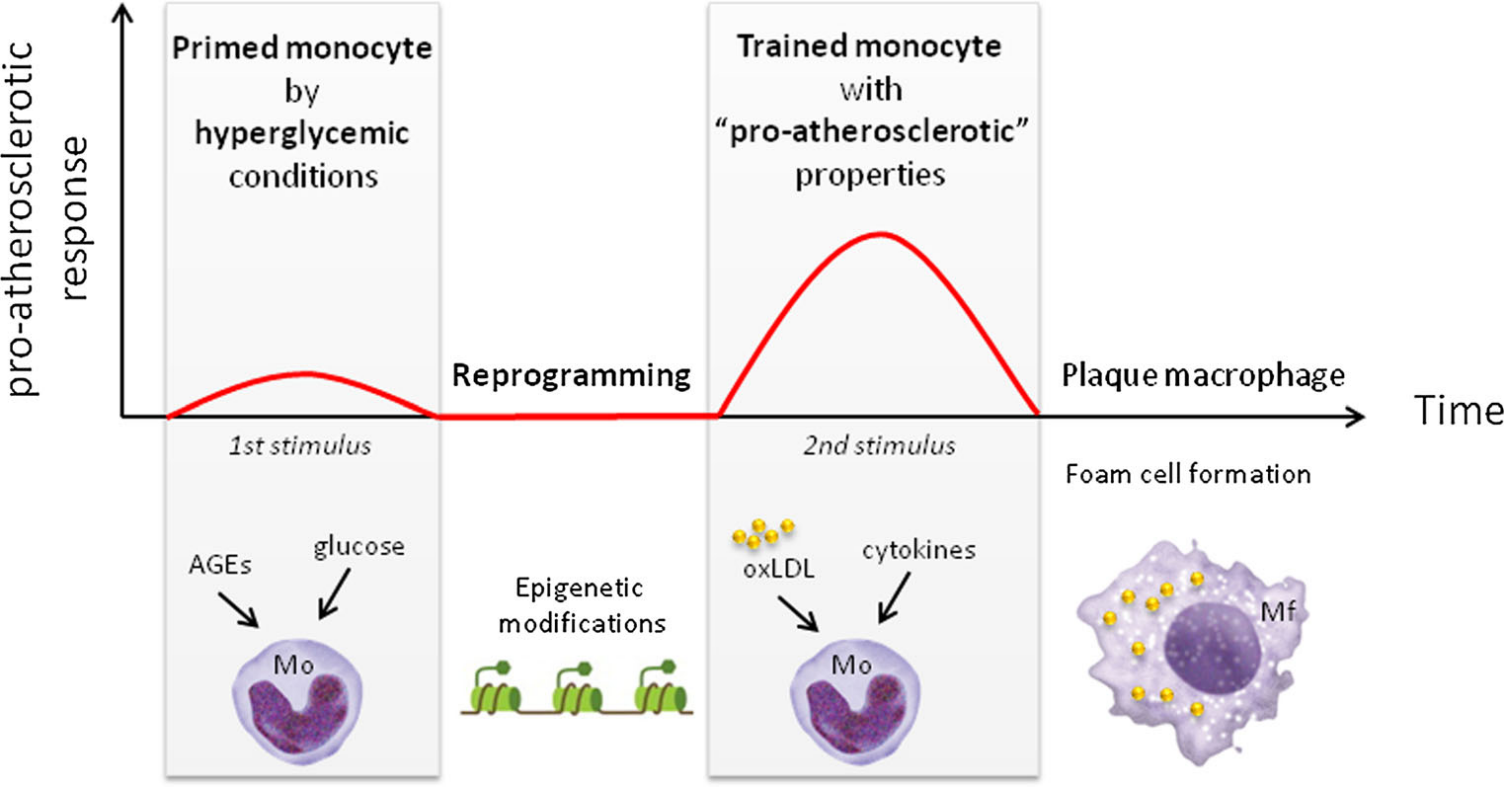
Schematic representation of the concept that hyperglycemia induces ‘trained immunity’ in monocytes. Initial stimulation of monocytes by hyperglycemic conditions (i.e., glucose or AGEs) induces epigenetic reprogramming of monocytes. The reprogramming of monocytes results in a long-term activated phenotype and increased response to a subsequent stimulus (e.g., oxLDL or cytokines). The more ‘aggressive’ proatherosclerotic response is characterized by, e.g., increased proinflammatory cytokine secretion and foam cell formation. *AGE* advanced glycation end products, *Mf* macrophage, *Mo* monocytes, *oxLDL* oxidized LDL

We hypothesize that hyperglycemic conditions in diabetes patients induces training of monocytes, functionally reprogramming the monocytes to display a more ‘aggressive’ proatherosclerotic response to subsequent encounter with other stimuli (e.g., cytokines and oxLDL). We propose that this process contributes to the phenomenon of hyperglycemic memory, resulting in enhanced plaque development and subsequent macrovascular complications (Fig. 1). Endogenous ‘training’ stimuli in diabetes patients, as well as mechanisms that confer the cell memory are so far unknown and remain to be established. In the remainder of this review, we discuss stimuli and pattern recognition receptors that may be involved in reprogramming of monocytes in patients with diabetes. In addition, we describe epigenetic changes that have been observed in monocytes from diabetes patients, and possibly contributed to proatherosclerotic reprogramming of these monocytes. Finally, we describe immunometabolic processes that are activated in trained innate immune cells, and may contribute to training of cells by hyperglycemic conditions.

### Training stimuli and pattern recognition receptors

Activation of recognition receptors on the surface of innate immune cells is likely to play a central role in innate immune memory [53, 54]. Several pattern-recognition receptors [PRRs; e.g., Toll-like receptors (TLRs) and C-type lectin receptors (CLRs)] of the innate immune system are involved in translating pattern-associated molecular patterns and (endogenous) danger signals to cellular responses, including training of monocytes [14]. Specific activation of the CLR Dectin-1 is involved in training of monocytes, resulting in enhanced proinflammatory cytokine secretion and foam cell formation [42, 48]. Multiple innate immune receptors including TLR2, TLR4, Mincle, NOD1/2, and NLRP3 are implicated in the recognition of dietary factors and endogenous danger signals, which translates into innate immune receptor signaling and inflammation in response to metabolic stress [55–57]. For example, free fatty acids are known to enhance TLR signaling, leading to activation of proinflammatory pathways [49]. CLRs are much less studied in relation to ‘metabolic inflammation’. CLRs are transmembrane receptors characterized by the presence of a carbohydrate-binding domain and highly expressed by macrophages. Macrophage-inducible CLRs such as Mincle or C-type Lectin 4e (Clec4e) recognize endogenous ligands from dying adipocytes and affect macrophage function in adipose tissue [56]. CLRs recognize carbohydrate structures present on pathogens [58]. It could be hypothesized that CLRs may also be able to recognize similar carbohydrate structures on endogenous (so far unknown) ligands in hyperglycemic/diabetic patients, such as AGEs. Within this respect, it is interesting that expression of the CLR Dectin-1 is increased on circulating monocytes from patients with diabetes as compared to healthy controls [59].

Collectively, PRRs and especially CLRs could perhaps translate hyperglycemic conditions into an elevated risk for the development of atherosclerosis via induction of trained immunity in monocytes. Involvement of individual PRRs, such as Dectin-1, as well as potential endogenous ligands that are known to be present under hyperglycemic conditions, such as AGEs, needs further evaluation.

### Epigenetic modifications

Immunological studies have shown that long-term reprogramming of monocytes is mediated by epigenetic changes [42, 43]. Whether hyperglycemia induces similar “training” and epigenetic changes in innate immune cells driving atherosclerotic development remains to be established. In mice, it has been shown that transient hyperglycemia induces H3K4me1 and reduces H3K9 demethylation in the promoter of the NF-κB in aortic endothelial cells, which associated with increased expression of monocyte chemoattractant protein-1 (MCP-1) and vascular cell adhesion molecule 1 (VCAM-1) and persisted for at least 6 days in vitro [60, 61]. In addition, other cell types, including vascular smooth muscle, retinal, and cardiac cells, respond to prior episodes of hyperglycemia with epigenetic changes [12, 13, 62]. Thus, strong evidence exists that hyperglycemic conditions can induce epigenetic reprogramming that alters expression of genes involved in the pathophysiology of diabetes complications [11, 12]. There are a few studies in primary monocytes derived from diabetes patients at risk for long-term complications. The first evidence for chromatin rearrangements in monocytes of diabetes patients came from Miao et al. showing increased histone H3 lysine 9 (H3K9) acetylation at the TNF and COX-2 promoter in monocytes from T1D and T2D patients [63]. Using chip-on-chip analysis, they additionally identified changes in lysine methylation [histone H3 lysine 4 dimethylation (H3K4me2) and histone H3 lysine 9 dimethylation (H3K9me2)] in proinflammatory genes in THP-1 monocytes induced by high glucose [63]. The first evidence for the presence of epigenetic histone posttranslational modifications in monocytes of diabetes patients comes from analysis of monocytes isolated from uncontrolled versus controlled T1D patients, a subgroup of patients from the DCCT/EDIC trial. Monocytes from uncontrolled conventionally treated subjects (>9.1 % Hba1c during DCCT) versus intensively treated subjects (<7.3 % HbA1c during DCCT) were recently assessed for H3K9Ac, H3K4Me3, and H3K9Me2. Importantly, monocytes from uncontrolled type 1 diabetes patients showed an increased acetylation of H3K9Ac in promoters of genes related to inflammatory pathways [64]. In addition, the methyltransferase Set7 may play a role in epigenetic regulation of NF-κB under hyperglycemic conditions, since Set7 is upregulated in monocytes from T2D and associates with fasting plasma glucose and HbA1c, as well as H3K4m1 of the NF-κB promoter region and expression of NF-κB target genes [65]. Evidence for persistence of epigenetic changes over time has only recently been shown by measuring epigenetic DNA methylation alterations in whole blood derived from a DCCT/EDIC subcohort, while comparing genomic DNA from intensively and conventionally treated patients before and after the EDIC follow-up [66]. Indeed, common differentially methylated loci were found, including changes in thioredoxin-interacting protein (TXNIP) known to be associated with hyperglycemia [66].

While there is evidence that certain epigenetic changes occur in monocytes of patients with diabetes and can persist over time, it remains to be proved that these are causally related to a proinflammatory, proatherogenic phenotype of monocytes, and subsequent cardiovascular complications. Moreover, the mechanisms by which epigenetic changes are induced in monocytes exposed to high glucose levels remain to be established.

### Intracellular metabolism

Recent interest and research focus on the direct relation between metabolism and chromatin dynamics [67], which would be an interesting concept to study in the context of epigenetic changes induced by high glucose exposure, especially because recent studies revealed that intracellular metabolism of glucose highly determines the activation status of innate immune cells during acute activation [68] as well as long-term activation of monocytes via trained immunity [45]. Upon an acute inflammatory stimulus, the cellular metabolism of an immune cell undergoes profound changes [69]. More specifically, whereas activation of glycolysis is associated with proinflammatory status of an immune cell, oxidative phosphorylation is mostly associated with an anti-inflammatory profile. Assuming that chronic hyperglycemia increases glucose availability as a substrate for innate immune cells, it could be hypothesized that high circulating glucose levels stimulate glucose utilization via glycolysis, thereby reprogramming innate immune cells toward a more proinflammatory phenotype. Indeed in vitro, the absence of the glucose transporter 1 (GLUT1) reduces both glycolysis and proinflammatory cytokine secretion [70, 71]. Vice versa, increased glycolysis induced by overexpression of GLUT1 leads to enhanced proinflammatory cytokine secretion [70]. However, in an experimental model in vivo, the increased glucose uptake did not stimulate inflammatory activation of peritoneal cells upon ex vivo stimulation with LPS, neither did it affect the development of atherosclerosis in Ldlr^−/−^ mice in vivo [71], suggesting that increased glucose supply alone is not sufficient to drive inflammatory activation and atherosclerosis in non-activated myeloid cells [72]. Possibly, synergism of glycolysis with other metabolic or immunologic pathways is necessary to induce the inflammatory phenotype in monocytes.

Recent data show that monocytes and macrophages of patients with atherosclerotic coronary artery disease display overutilization of glucose, promoting excessive and prolonged production of proinflammatory cytokines [52]. These data support the hypothesis that increased utilization of glucose is involved in reprogramming of innate immune cells toward a proinflammatory, proatherogenic phenotype. Whether this is exacerbated under hyperglycemic conditions is so far unknown and requires further investigation. Nevertheless, reducing glycolysis corrects the proinflammatory phenotype of macrophages derived from patients with atherosclerotic coronary artery disease [52]. In addition, a reduction in glycolysis in myeloid cells via GLUT1 deletion inhibits expansion and proliferation of myeloid progenitor cells, leading to a reduction of plaque macrophages in an animal model for atherosclerosis in vivo [73].

An important molecular switch that drives cellular metabolism in macrophages is AMP-activated protein kinase (AMPK) [68]. Importantly, activation of AMPK has been demonstrated to exert anti-inflammatory effects [68]. Metformin is a glucose lowering drug that activates AMP-activated protein kinase (AMPK) in vitro and reduces redox shuttle enzyme mitochondrial glycerophosphate dehydrogenase [74, 75]. Metformin has been shown to inhibit the process of trained immunity induced by β-glucan [45]. Interestingly, metformin treatment of T2D patients increases AMPK activation in monocyte-derived macrophages [24]. Moreover, metformin treatment reduced T2D monocyte activation, measured by a reduction in inflammasome activation and IL-1β secretion ex vivo [24]. These data suggest that metformin can reduce ‘training’ of monocytes that has initially been induced by a diabetic environment. Such properties may contribute to the demonstrated beneficial effects of metformin on cardiovascular outcomes in patients with diabetes.

In sum, the long-term reprogramming of monocytes by pathogenic stimuli involves a shift in cellular metabolism from oxidative phosphorylation to aerobic glycolysis. We hypothesize that hyperglycemia in diabetes patients induces long-term activation of monocytes and macrophages through similar mechanisms, thereby contributing to plaque development and subsequent macrovascular complications.

## Concluding remarks and future directions

Chronically elevated glucose levels are one of the main risk factors for CVD, rendering blood glucose control of utmost importance. However, the hyperglycemic memory induced by previous episodes of uncontrolled blood glucose levels limits subsequent effectiveness of glucose control to prevent long-term CVD complications. High glucose levels might prime innate immune cells and change their epigenetic profile to a predisposed proinflammatory state, referred to as “trained immunity” ultimately promoting atherosclerotic plaque development. Effectiveness of glucose lowering therapies in diabetes patients may suffer from such training effects, reducing effectiveness because of an imprinted proinflammatory response.

While compelling new data suggest that this effect of hyperglycemia on long-term functional reprogramming of monocytes and macrophages is important in the pathophysiology of atherosclerosis in patients with diabetes, many crucial factors remain to be explored. First, the life span of circulating monocytes ranges from days to weeks, while deleterious effects of exposure to hyperglycemia persist for years after implementation of improved glycemic control. It may well be that elevated glucose levels already induce ‘training’ and (epigenetic) changes in hematopoietic stem cells (HSCs) within the bone marrow. Recent evidence from diabetes animal models characterized by high glucose levels indeed shows alterations in HSCs—including epigenetic changes and a prolonged intrinsic proinflammatory phenotype [23, 76, 77]. Other factors that remain to be explored are the causality between specific epigenetic changes and the functional and clinical complications; the potential roles of epigenetic mechanisms additional to chromatin structure and histone modifications such as long non-coding RNAs; the molecular switches leading to these changes, and identification of novel strategies to correct the long-term immune activation to improve the outcome of the patients. Only when these steps have been undertaken, the full impact of these discoveries becomes available.

## Abbreviations

AGE: Advanced glycation end product
AMPK: AMP-activated protein kinase
BCG: Bacillus Calmette–Guérin
CLR: C-type lectin receptor
CVD: Cardiovascular diseases
DCCT/EDIC: Diabetes Control and Complications Trial and Epidemiology of Diabetes Interventions and Complications study
FFA: Free fatty acid
GLUT1: Glucose transporter 1
H3K4me1: Histone H3 lysine 4 monomethylation
H3K4me3: Histone H3 lysine 4 trimethylation
H2K27Ac: Histone H2 lysine 27 acetylation
H3K9me2: Histone H3 lysine 9 dimethylation
HbA1c: Hemoglobin A1c
IL-6: Interleukin 6
LPS: Lipopolysaccharide
MCP-1: Monocyte chemoattractant protein-1
NF-κB: Nuclear factor κB
oxLDL: Oxidized low density lipoprotein
PMA: Phorbol 12-myristate 13-acetate
ROS: Reactive oxygen species
PRR: Pattern recognition receptor
TLR: Toll-like receptor
T1D: Type 1 diabetes
T2D: Type 2 diabetes
TNFα: Tumor necrosis factor α
TXNIP: Thioredoxin-interacting protein
UKPDS: United Kingdom Prospective Diabetes Study
VCAM-1: Vascular cell adhesion molecule 1
